# Association of Oxytocin Receptor Gene (OXTR) rs53576 Polymorphism with Sociality: A Meta-Analysis

**DOI:** 10.1371/journal.pone.0131820

**Published:** 2015-06-29

**Authors:** Jingguang Li, Yajun Zhao, Rena Li, Lucas S. Broster, Chenglin Zhou, Suyong Yang

**Affiliations:** 1 College of Education, Dali University, Dali, China; 2 College of Sociology and Psychology, Southwest University for Nationalities, Chengdu, China; 3 Center for Hormone Advanced Science and Education, Roskamp Institute, Sarasota, Florida, United States of America; 4 Key Laboratory of Exercise and Health Sciences of Ministry of Education, Shanghai University of Sport, Shanghai, China; 5 Department of Behavioral Science, University of Kentucky, Lexington, Kentucky, United States of America; University of Chicago, UNITED STATES

## Abstract

A common variant in the oxytocin receptor gene (OXTR), rs53576, has been broadly linked to socially related personality traits and behaviors. However, the pattern of published results is inconsistent. Here, we performed a meta-analysis to comprehensively evaluate the association. The literature was searched for relevant studies and effect sizes between individuals homozygous for the G allele (GG) and individuals with A allele carriers (AA/AG). Specifically, two indices of sociality were evaluated independently: i) general sociality (24 samples, n = 4955), i.e., how an individual responds to other people in general; and ii) close relationships (15 samples, n = 5262), i.e., how an individual responds to individuals with closed connections (parent-child or romantic relationship). We found positive association between the rs53576 polymorphism and general sociality (Cohen’s *d* = 0.11, *p* = .02); G allele homozygotes had higher general sociality than the A allele carriers. However, the meta-analyses did not detect significant genetic association between rs53576 and close relationships (Cohen’s *d* = 0.01, *p* = .64). In conclusion, genetic variation in the rs53576 influences general sociality, which further implies that it is worthy to systematically examine whether the rs53576 is a valid genetic marker for socially related psychiatric disorders.

## Introduction

Social interaction is important to almost every aspect of human life. However, people differ in their degree of sociality. Whereas some people enjoy an active social life and easily interacting with others, some people avoid social interactions and have difficulty in dealing with people. Extreme examples of the latter condition include several socially related psychiatric disorders, including autism and social anxiety disorder. Understanding the molecular genetic basis of individual differences in sociality has received significant research interest of late [1,2].

Previous studies have implied that the oxytocin system plays an important role in human socially related personality traits and behaviors [3,4], hereafter referred to as “sociality.” For example, high levels of plasma oxytocin (OXT) have been associated with behaviors indicative of enhanced sociality, such as increased physical contact with a partner [5] and trustworthiness [6]. Moreover, intranasal administration of OXT can specifically enhance sociality as well, by increasing memory for social information [7], trust [6] and altruistic behaviors [8].

Due to the preservation of the oxytocin neuropeptide across mammalian species and the heritability of sociality in humans [9], variations in genes encoding oxytocin may account for individual differences in sociality [4]. The oxytocin receptor gene (OXTR), located on chromosome 3p25, is one such candidate. Indeed, several studies have examined the link between the OXTR polymorphism and sociality. Compared with other single nucleotide polymorphisms (SNPs) in OXTR, one SNP in the third intron of OXTR, rs53576 (G/A), has received the most attention. Compared with A allele carriers (AA/AG), recent studies have found that the G allele homozygotes (GG) were associated with high sociality, such as self-reported empathy [10], dependence on social reward [11], social auditory ability [12], and general sociality as rated by peers [13]. However, like many reported genetic associations, not all of these findings have been successfully replicated [14,15].

Using a meta-analysis approach, a recent study conducted by Bakermans-Kranenburg and van IJzendoorn attempted to evaluate the association between the rs53576 polymorphism and sociality and did not establish a statistically significant genetic association [16]. However, we argue that the results of that meta-analysis might be inconclusive in that the researchers might have inadvertently conflated general sociality with a particular subtype of sociality–that is, sociality in the context of close relationships [17]. Here, we define close relationships in the context of the most important evolutionary purpose–reproduction. Thus, close relationships in our study specifically correspond to three examples, i.e., relationships with a caregiver (i.e., father or mother), one’s own child, or a romantic partner. However, other measures of sociality (e.g., extraversion, empathy, and trust) often involve how an individual responds to other people in general, which involves not only caregivers, children, and partners, but also friends, teachers, colleagues, and even strangers. According to evolutionary theory, our mind has evolved specific mechanisms in service of social situations relevant to reproduction [18]. Therefore, given the unique adaptive problems each type of response is intended to solve, individuals’ responses to close individuals may differ considerably relative to their responses to others. This contention has been supported by abundant psychological literature [17, 19]. For example, infants’ responses to their mothers differ considerably relative to those to strangers [20]. We suggest that the genetic underpinning between these two psychological measures might be distinct. In such a case, a comprehensive and appropriate evaluation of the association between rs53576 polymorphisms and sociality would require separate meta-analyses for, at the very least, general sociality and sociality in the context of close relationships.

In this study, we reexamined the association between rs53576 polymorphisms and sociality in humans with a meta-analytic approach. Compared with the previously mentioned meta-analysis [16], our study differed in three aspects. First, and most critically, we performed separated analysis on general sociality and close relationship. Second, withdrawal from social situations and activities is often associated with depression [21], and a study has reported genetic associations between rs53576 polymorphism and depression [22] (see also [23] for a narrative review on this issue).we assessed the possibility that genetic associations between rs53576 polymorphism and sociality might be mediated by depression. Specifically, we will first examine whether rs53576 polymorphism is related to individual differences in depression; if positive association was detected, we will then examine the possible mediation relationship [24]. Third, compared to Bakermans-Kranenburg and van IJzendoorn’s meta-analysis, we drew from an updated database of relevant genetic association studies such that 7 additional empirical studies consistent of 12 independent samples were assessed [12,25–30].

## Materials and Methods

### Literature search

To identify eligible studies for this meta-analysis, we searched the PubMed and ISI Web of Knowledge for all publications available up to May 1st, 2014 that examined the association between the OXTR rs53576 polymorphism and psychological traits. The following terms were included in the search: oxytocin receptor (or OXTR) and gene. In addition, we identified additional relevant studies from the references of initially identified studies and recent literature reviews.

### Selection of phenotype and data extraction

We first identified all candidate measures from studies that examined the rs53576 polymorphism and psychological traits. Then, among all the psychological measures, phenotypes relating: 1) general sociality (e.g., extraversion, empathy, and social loneliness); 2) close relationship (including maternal sensitivity, child/adult attachment, and marital quality); and 3) depression were selected and classified into three independent categories respectively.

Data were extracted by the first and last author of the current manuscript independently and then cross-checked. In addition, when a sample consisted of more than one eligible phenotype, the phenotype that appeared most frequently in the literature was selected. If the relevant data were not reported in the article, the authors were contacted and requested for data releasing. 22 out of 27 authors released their data in an appropriate format. One data point (i.e., effect size) was extracted from one sample.

For each sample, the following information was extracted: first author, year of publication, country and ethnicity of the participants, proportion of females, diagnostic status (i.e., healthy individuals or patients), and age. Reverse scoring was used as necessary to maintain consistent measurement scaling across studies.

### Data analysis

Following the convention in most published studies, OXTR rs53576 genotypes were grouped according to the presence and absence of the A allele (GG vs. GA+AA) [10,11,13,31]. We performed the analysis using the Metafor package in R [32]. Cohen’s *d* was used as the effect size measure, representing the standardized mean difference. Because the heterogeneous nature of included studies (e.g., differences in tasks to measure sociality), the weighted effect size and 95% confidence interval

(95% CI) were generated via a random-effects analysis model by DerSimonian-Laird estimators [33]. In addition, the Q-profile method was used to confirm the heterogeneity of the included studies [34]. In the random-effects analysis model, between-study heterogeneity is proposed to result from both random variation and effects from individual studies. Random-effects models are generally more conservative (i.e., narrower CI) than fixed-effect models. Finally, *Z* scores were used to assess the statistical significance of the weighted effect size.

Publication bias was assessed by the Begg’s Test and the Funnel plot in order to assess the tendency to publish positive results rather than negative results. A sensitivity analysis was applied to examine whether the inclusion of a particular study had a significant effect on the overall weighted effect size. That is, studies were removed one at a time to examine whether the pooled effect size remained statistically significant or non-significant. Finally, to examine the potential influence of any moderator variables, we performed meta-regression analyses with the average age of the sample, proportion of female participants, and proportion of Caucasian participants in the sample as predictors. Because not all studies reported demographic information, not all samples were included in the moderation analyses.

## Results

**Fig 1:**
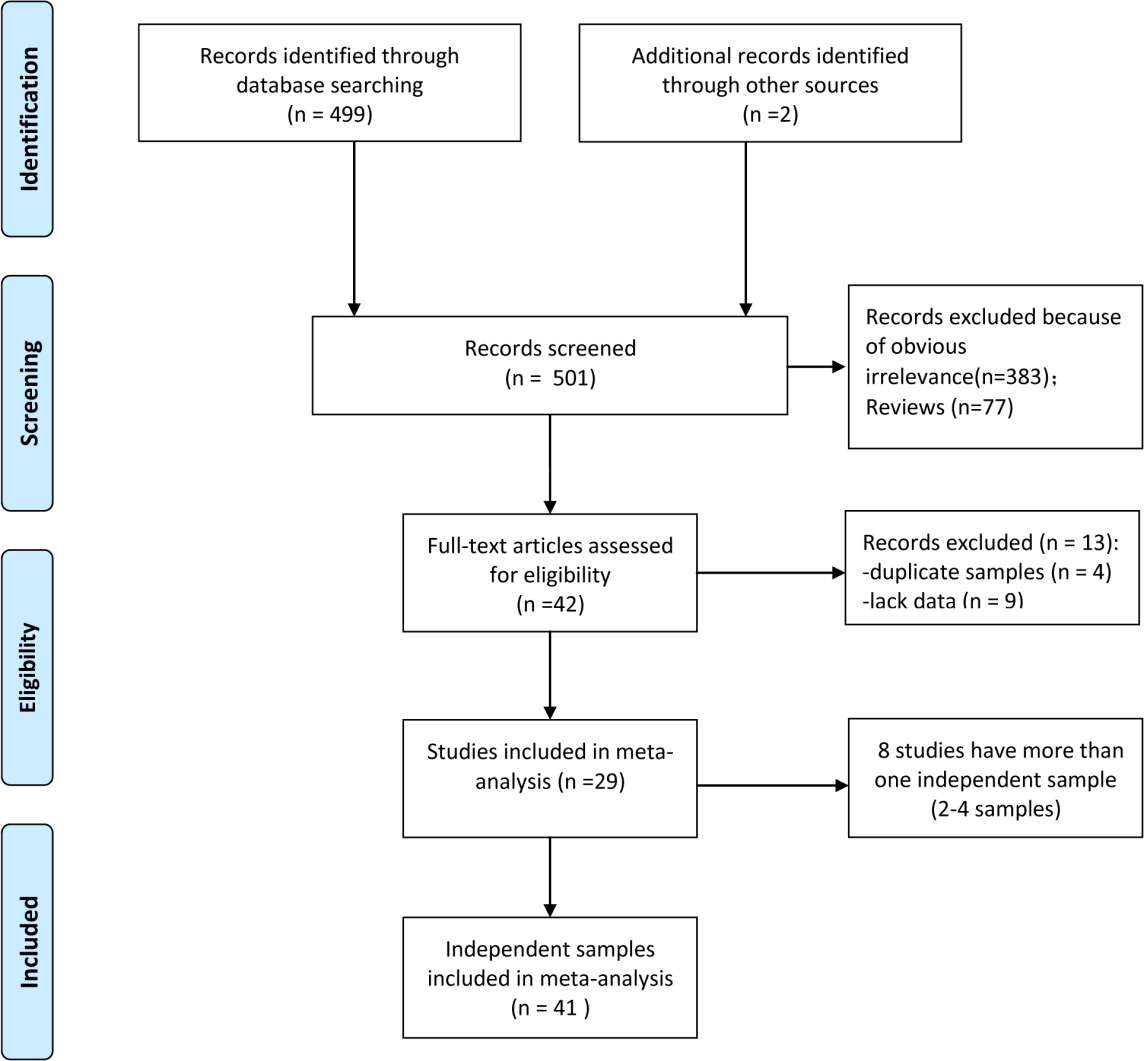
Flow chart of study selection in the meta-analysis.

Our meta-analysis was performed according to two guidelines: the “Preferred Reporting Items for Systematic Reviews and Meta-Analyses” (PRISMA) statement (S1 Table) [35] and the “Meta-analysis on Genetic Association Studies” statement (S2 Table) [36]. Fig 1 shows the flow chart of the literature search process. The excluded articles and reasons for exclusion were lists in the Supporting Information (S1 File). After the literature search was completed, we conducted separated meta-analyses on the associations between the rs53576 polymorphism and three phenotype categories (i.e., general sociality, close relationship, and depression).

### General sociality and close relationship

**Fig 2:**
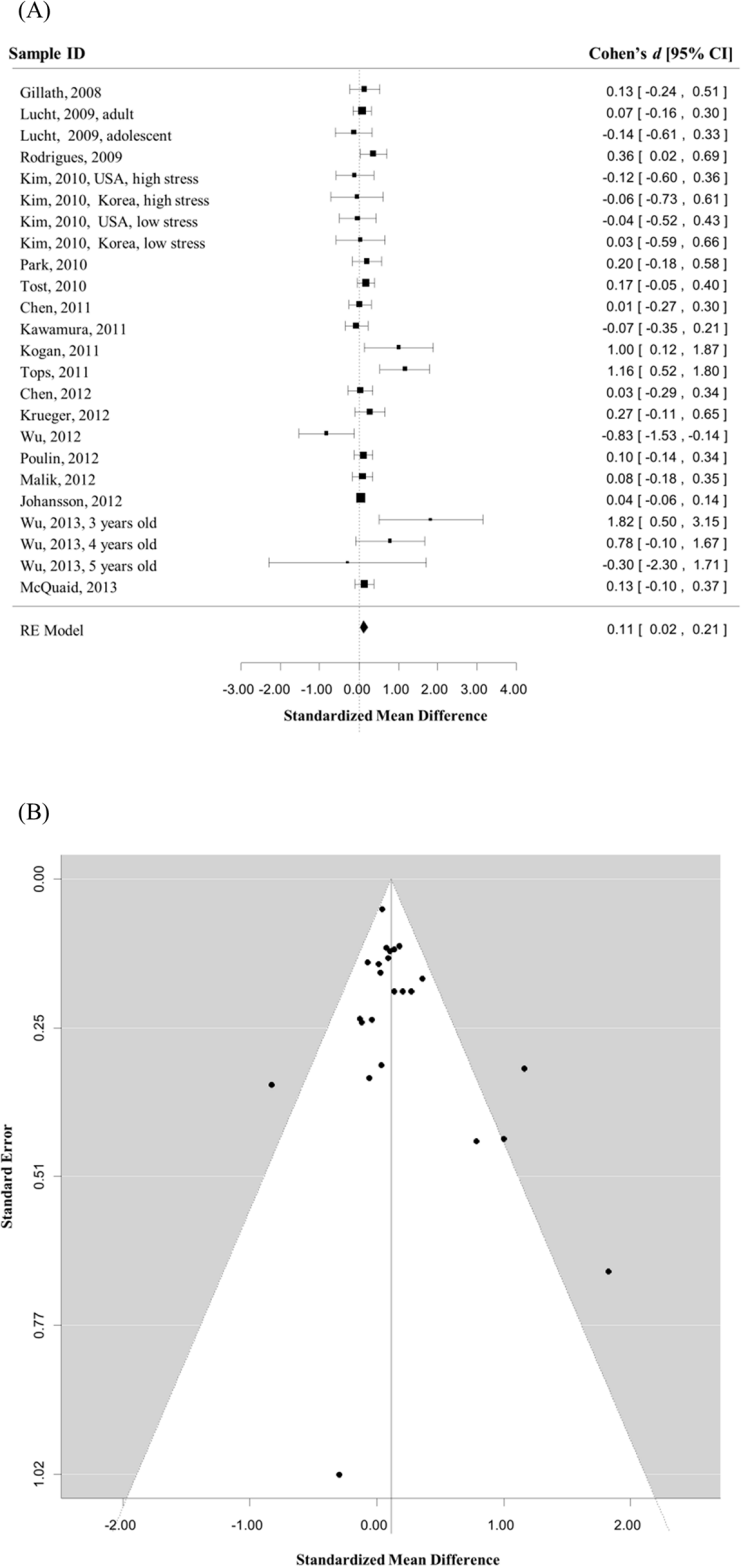
The association between rs53576 polymorphism and general sociality. A) Magnitudes of effect size for the association between rs53576 polymorphism and general sociality are illustrated by the forest plot (AA + GA vs. GG). Boxes represent the effect size (Cohen’s *d*) for each sample in the analysis; the size of the boxes represents the weighting for each study; lines represent the .95 confidence interval for each effect size; and the diamond represents the overall effect of the meta-analysis, which was obtained by a random effects model. B) Publication bias is illustrated by the funnel plot. The horizontal axis represents the effect size of each study. The vertical axis represents the size of each study (indexed by the standard error of the effect size within each study). In consequence, large studies appear towards the top of the graph and small studies appear towards the bottom of the graph. A vertical line indicates the estimated overall effect size. A confidence interval region is drawn around this value with bounds equal to ± 1.96 standard error. In the absence of publication bias, the studies will be distributed symmetrically around the vertical line. Otherwise, the studies will be distributed asymmetrically.

Eighteen studies [10–15,25,27,30–31,37–44] comprising twenty-four independent samples (Table 1) contributed to the meta-analysis of general sociality (total number of participants = 4955). As expected, there was evidence of between-study heterogeneity (*Q* = 40.08, *p* = .02). Critically, the random effects analysis indicated an association (Cohen’s *d* = 0.11, 95% CI = [0.02, 0.21], *Z* = 2.28, *p* = .02) such that G allele homozygotes had higher general sociality than A allele carriers (Fig 2A). The Begg’s test indicated no evidence of publication bias (Kendall's tau = 0.09, *p* = .54) (Fig 2B). Because the sensitivity analysis indicated that pattern of results was similar after removal of any individual sample from the meta-analysis (i.e., all Cohen’s *d*s > = 0.09, *p*s < .05), the association observed in the meta-analysis was considered unlikely to be accounted for by any single outlying sample. Finally, meta-regression analyses suggested that the association was not significantly affected by sex (number of studies included (*k*) = 24, *p* = .20), age (*k* = 21, *p* = .31), or ethnicity (*k* = 24, *p* = .48).

**Table 1:** Characteristics of studies on the association between rs53576 polymorphism and sociality.

Authors (Year)	Country	Ethnicity	% of Female	Diagnosis	Age (Mean/SD)	Phenotypes	Measurement tools
Gillath et al., 2008 [38]	U.S.A.	31% Caucasian & 44% Asian	73%	Healthy	Range: 18–29	Extraversion	Big Five Personality Inventory
Lucht et al., 2009, adult [14]	Germany	N.A.	65%	Healthy	41.7 (7.2)	Social loneliness	UCLA Loneliness Scale
Lucht et al., 2009, adolescent [14]	Germany	N.A.	50%	Healthy	15.1 (2.1)	Social loneliness	UCLA Loneliness Scale
Rodrigues et al., 2009 [10]	U.S.A.	35% Caucasian & 41% Asian	59%	Healthy	20.2 (N.A.)	Empathy	Interpersonal Reactivity Index
Kim et al., 2010, High stress [25]	U.S.A.	77% Caucasian & 23% Asian	56%	Healthy	24.5 (N.A.)	Emotional support seeking	The COPE Inventory: Emotion Coping Subscale
Kim et al., 2010, High stress [25]	Korean	100% Asian	47%	Healthy	25.1 (N.A.)	Emotional support seeking	The COPE Inventory: Emotion Coping Subscale
Kim et al., 2010, Low stress [25]	U.S.A.	77% Caucasian & 23% Asian	56%	Healthy	24.5 (N.A.)	Emotional support seeking	The COPE Inventory: Emotion Coping Subscale
Kim et al., 2010, Low stress [25]	Korean	100% Asian	47%	Healthy	25.1 (N.A.)	Emotional support seeking	The COPE Inventory: Emotion Coping Subscale
Park et al., 2010 [43]	UK & Ireland	100% Caucasian	10%	ADHD	Range: 4–16	Autistic traits	Social and Communication Disorder Checklist
Tost et al., 2010 [11]	U.S.A.	100% Caucasian	53%	Healthy	30.8 (9.2)	Reward dependence	Tridimensional Personality Questionnaire
Chen et al., 2011 [31]	Germany	89% Caucasian	0%	Healthy	23.2 (2.9)	Empathy	Interpersonal Reactivity Index
Kawamura et al., 2011 [40]	Japan	100% Asian	38%	Healthy	40.9 (9.7)	Social skill	Autism Spectrum Quotient: Social Skill Subscale
Kogan et al., 2011 [13]	U.S.A.	100% Caucasian	48%	Healthy	23.8 (3.5)	Nonverbal cues of sociality	Laboratory Observation
Tops et al., 2011 [12]	Netherlands	94% Caucasian	100%	Healthy	29 (7.4)	Social auditory ability	Self-Reported Social Auditory Ability
Chen et al., 2012 [37]	U.S.A.	42% Caucasian & 28% Asian	61%	Healthy	N.A.	Social skill	Autism Spectrum Quotient: Social Skill Subscale
Krueger et al., 2012 [41]	U.S.A.	100% Caucasian	0%	Healthy	20.2 (2.2)	Empathy	Interpersonal Reactivity Index
Wu et al., 2012 [15]	China	100% Asian	54%	Healthy	22.5 (2.3)	Empathy	Interpersonal Reactivity Index
Poulin et al., 2012 [44]	U.S.A.	100% Caucasian	51%	Healthy	N.A.	Civic duty	Social And Political Survey
Johansson et al., 2012 [39]	Finland	100% Caucasian	58%	Healthy	26.4 (4.8)	Aggressive behavior	Buss and Perry Aggression Questionnaire
Malik et al., 2012 [42]	Canada	82% Caucasian	31%	Antisocial	11. 5 (3.0)	Aggressive traits	Achenbach Child Behaviour Checklist
Wu et al., 2013, 3-years old [30]	China	100% Asian	49%	Healthy	3	Prosocial behavior	Total scores of helping behavior, comforting behavior; sharing behavior
Wu et al., 2013, 4-years old [30]	China	100% Asian	49%	Healthy	4	Prosocial behavior	Total scores of helping behavior, comforting behavior; sharing behavior
Wu et al., 2013, 5-years old [30]	China	100% Asian	49%	Healthy	5	Prosocial behavior	Total scores of helping behavior, comforting behavior; sharing behavior
McQuaid et al., 2013 [27]	Canada	58% Caucasian	74%	Healthy	20.0(3.2)	Distrust and Cynicism	Distrust and Cynicism Scale

**Fig 3:**
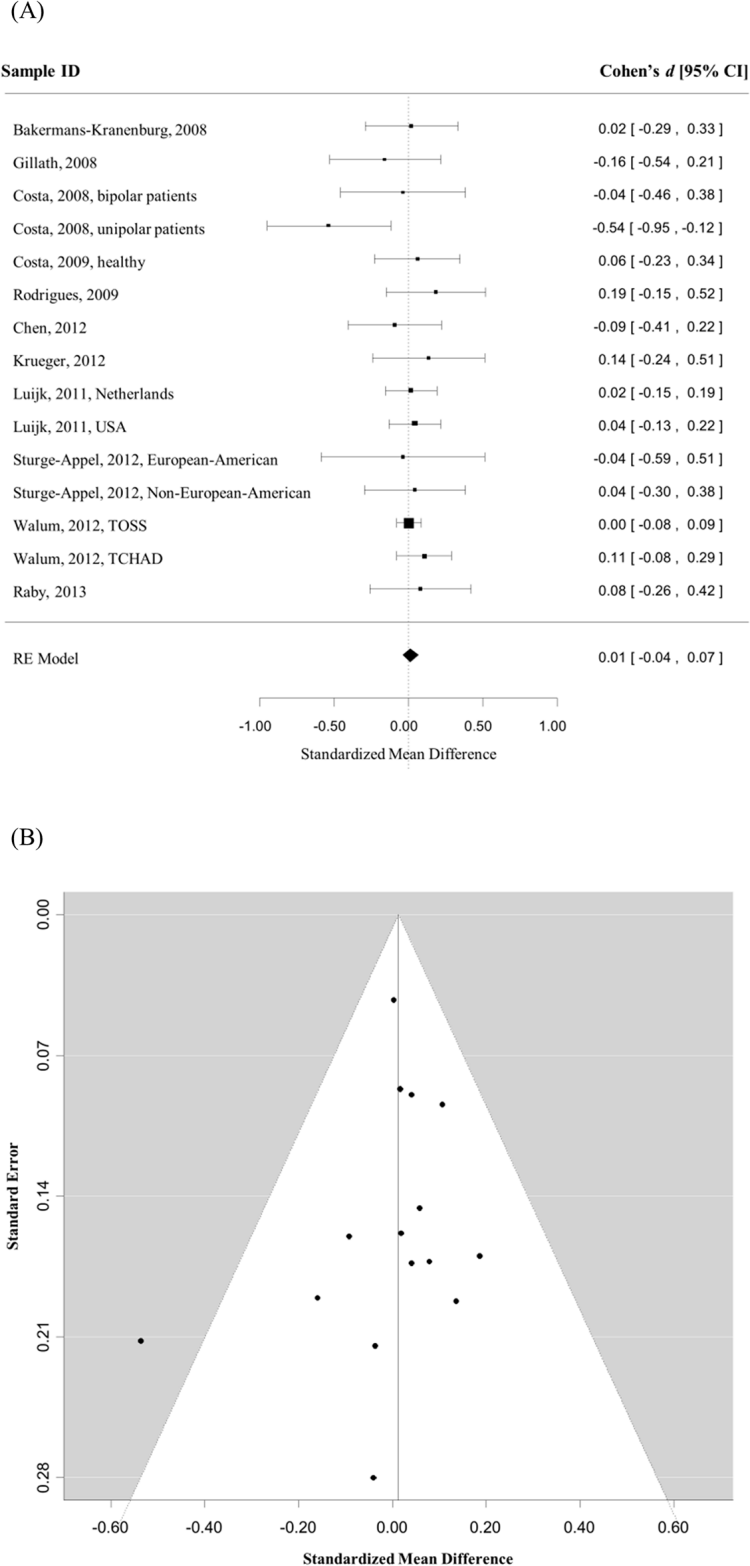
The association between rs53576 polymorphism and close relationship. A) Magnitudes of effect size for the association between rs53576 polymorphism and individual differences in close relationship are illustrated by the forest plot (AA + GA vs. GG). Boxes represent the effect size (Cohen’s *d*) for each sample in the analysis; the size of the boxes represents the weighting for each study; lines represent the .95 confidence interval for each effect size; and the diamond represents the overall effect of the meta-analysis, which was obtained by a random effects model. B) Publication bias is illustrated by the funnel plot. The horizontal axis represents the effect size of each study, and the vertical axis represents the size of each study (indexed by the standard error of the effect size within each study).

Next, we tested whether the rs53576 polymorphism influenced the quality of close relationship, a specific kind of sociality measurement. Ten studies [10,28,37,38,41,45–49] comprising 15 independent samples (Table 2) contributed to the meta-analysis (total n = 5262). Although there was no evidence of between-study heterogeneity (Q = 10.86, *p* = .70), we used a random-effects model in the following meta-analysis because the tasks measuring close relationships were subjectively disparate (c.f., Table 2, the last column). In contrast to the observed positive genetic association on general sociality, meta-analysis (GG vs. GA+AA) indicated no evidence of association for close relationship (*d* = 0.01, 95% CI = [-0.04, 0.07], *Z* = 0.47, *p* = .64) (Fig 3A). The Begg’s test (Kendall's tau = -0.14, *p* = .50) indicated no evidence of publication bias (Fig 3B), and the sensitivity analysis indicated that the lack of association was not due to any particular data point in the overall sample (all Cohen’s *d* < = 0.02, *p*s > .42). Finally, meta-regression analyses indicated that sex (k = 15, *p* = .41), age (k = 13, *p* = .42), and ethnicity (k = 15, *p* = .90) did not significantly influence the association.

**Table 2:** Characteristics of studies on the association between rs53576 polymorphism and close relationship.

Authors (Year)	Country	Ethnicity	% of Female	Diagnosis	Age (Mean/SD)	Phenotypes	Measurement tools
Bakermans-Kranenburg et al., 2008 [45]	Netherlands	95% Caucasian	100%	Healthy	33 (4.1)	Marital discord	Dutch Family Problems Questionnaire (subscale)
Gillath et al., 2008 [38]	U.S.A.	31% Caucasian & 44% Asian	73%	Healthy	Range: 18–29	Attachment anxiety	Experiences in Close Relationships Inventory
Costa et al., 2009 [48]	Italy	100% Caucasian	73%	Bipolar disorder	40.9 (11.7)	Separation anxiety	Adult Separation Anxiety Checklist
Costa et al., 2009 [48]	Italy	100% Caucasian	68%	Unipolar depression	44.4 (12.5)	Separation anxiety	Adult Separation Anxiety Checklist
Costa et al., 2009 [48]	Italy	100% Caucasian	69%	Healthy	42.2 (11.0)	Separation anxiety	Adult Separation Anxiety Checklist
Rodrigues et al., 2009 [10]	U.S.A.	35% Caucasian, 41% Asian	59%	Healthy	20.2 (2.8)	Attachment anxiety	Experiences in Close Relationships Inventory
Chen et al., 2012 [37]	U.S.A.	42% Causion, 28%Asian	61%	Healthy	N.A.	Attachment anxiety	Experiences in Close Relationships Scale
Krueger et al., 2012 [41]	U.S.A.	100% Caucasian	0%	Healthy	20.2 (2.2)	Attachment (secure)	Relationship Scale Questionnaire
Luijk et al., 2011 [46]	Netherlands	100% Caucasian	48%	Healthy	1.3	Attachment security	Strange Situation Procedure; Attachment Security Scale
Luijk et al., 2011 [46]	N.A.	100% Caucasian	52%	Healthy	1.3	Attachment security	Strange Situation Procedure; Attachment Security Scale
Sturge-Appel et al., 2012 [47]	U.S.A.	100% Caucasian	100%	Healthy	28.5 (6.0)	Interpartner Conflict	Conflict Tactics Scale 2; Conflict and Problem-Solving Scale
Sturge-Appel et al., 2012 [47]	U.S.A.	100% non-Caucasian	100%	Healthy	25. 5 (5.8)	Interpartner Conflict	Conflict Tactics Scale 2; Conflict and Problem-Solving Scale
Walum et al., 2012 (TOSS) [49]	Sweden	100% Caucasian	63%	Healthy	Range:32–74	Pair-bonding	Pair-bonding Scale
Walum et al., 2012 (TCHAD) [49]	Sweden	100% Caucasian	57%	Healthy	Range:19–20	Pair-bonding	Relationship Quality Survey (affection scale)
Raby et al., 2013 [28]	U.S.A.	66% Caucasian	50%	Healthy	26	romantic relationship security	Current Relationship Interview

In short, the aforementioned analyses revealed that rs53576 polymorphism explains the variances in general sociality, but not the variances in close relationship. This finding appears to be different from the findings of another meta-analytical study by Bakermans-Kranenburg and van IJzendoorn, who reported that no genetic association between rs53576 and sociality [16]. To explore possible reasons for this inconsistency, we compared the methodologies between two studies and performed additional analyses. The current meta-analysis differs from theirs in two major ways. First, we performed separate analyses on general sociality and close relationship, whereas Bakermans-Kranenburg and van IJzendoorn [16] did not distinguish these phenotypes. Second, the included empirical studies in the two meta-analyses were not identical. Because we perceived the theoretical significance of these contrasts between meta-analysis designs to differ (i.e., the separate analysis for distinct sociality phenotypes being more compelling than slight changes in the studies included), we repeated the previous analyses using only the studies that had also been used by Bakermans-Kranenburg and van IJzendoorn to try to rule-out the possibility that the inclusion of the additional studies were not responsible for the genetic association identified.

First, when general sociality and close relationships were categorized as a single phenotype, the random effects model indicated no evidence of genetic association between all sociality measures (*p*s > .10). Note that several studies simultaneously included measures of general sociality and intimate relationship [10,37,38,41]. Therefore, to ensure that each study contributed only one data point to each meta-analysis, we have performed two types of analyses. In one situation, only data about sociality were entered in the final analyses; in the other situation, only data about the close relationship were entered in the final analyses. Random effect analyses suggested that both the first (k = 25, Cohen’s *d* = 0.04, 95% CI = [-0.008, 0.097], Z = 1.65, *p* = .10) and second (k = 25, Cohen’s *d* = 0.03, 95% CI = [-0.02, 0.08], Z = 1.19, *p* = .23) analyses yielded statistically non-significant results. Second, when only data on general sociality were included, a trending genetic association was detected (k = 15, Cohen’s *d* = 0.08, 95% CI = [-0.002, 0.17], *Z* = 1.91, *p* = 0.06). Third, when data on close relationships were included, there was no evidence of genetic association (k = 14, Cohen’s *d* = 0.01, 95% CI = [-0.04, 0.07], *Z* = 0.41, *p* = 0.69).

Taken together, our meta-analysis included more empirical studies, which increased the statistical power of the meta-analysis and facilitated detection of the genetic association; however, the phenomenon of increased effect size when the data were separated according to general sociality and close relationships was maintained even when only literature included in the previous meta-analysis was evaluated.

### Depression

**Fig 4:**
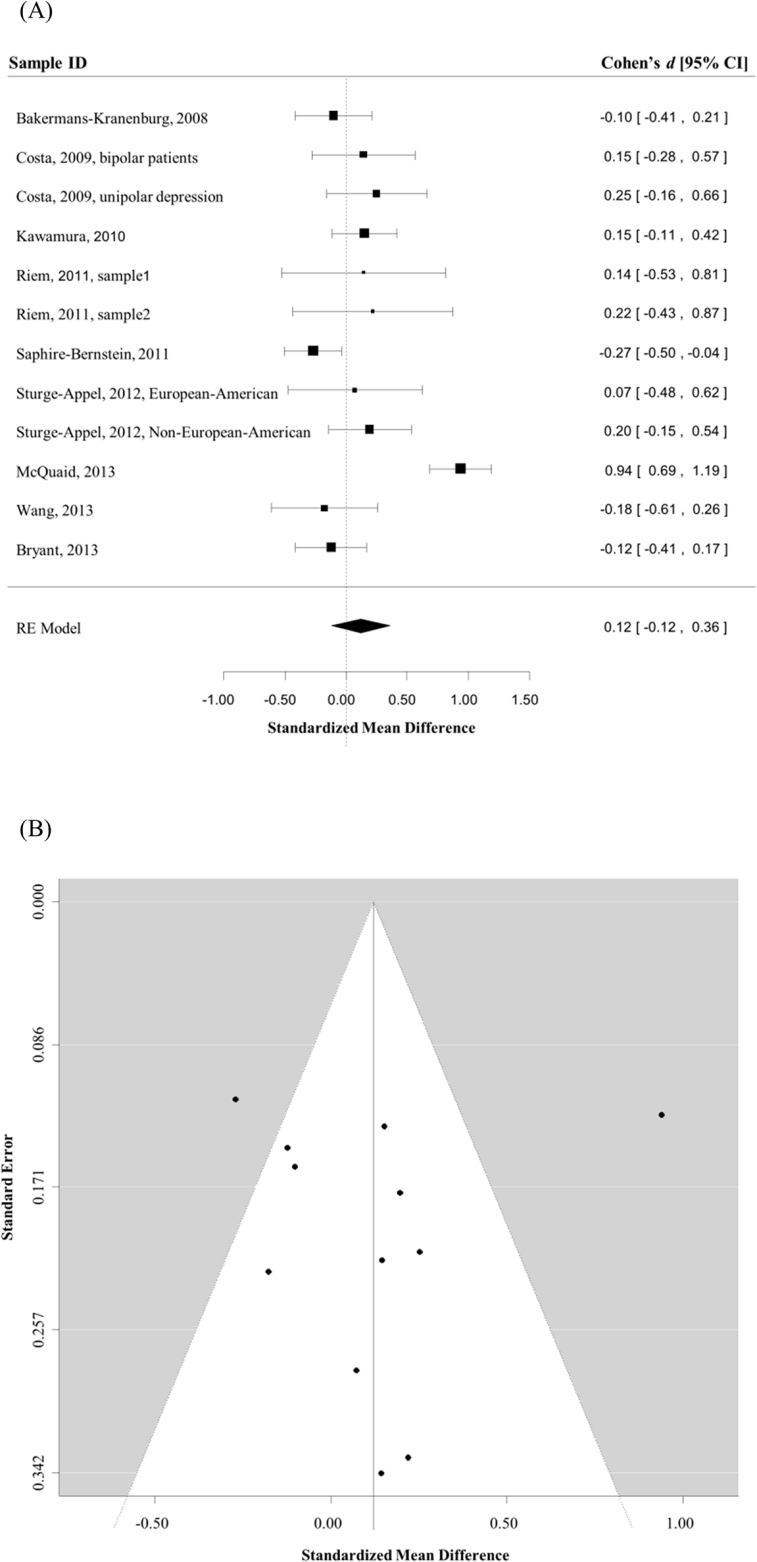
The association between rs53576 polymorphism and depression. A) Magnitudes of effect size for the association between rs53576 polymorphism and depression are illustrated by the forest plot (AA + GA vs. GG). Boxes represent the effect size (Cohen’s *d*) for each sample in the analysis; the size of the boxes represents the weighting for each study; lines represent the .95 confidence interval for each effect size; and the diamond represents the overall effect of the meta-analysis, which was obtained by a random effects model. B) Publication bias is illustrated by the funnel plot. The horizontal axis represents the effect size of each study, and the vertical axis represents the size of each study (indexed by the standard error of the effect size within each study).

As we noted previously, depression and lack of sociality are often related [21]. Therefore, the association between rs53576 polymorphism and general sociality might be mediated by depression. To examine this possibility, we also examined the association between the rs53576 polymorphism and depression using meta-analysis. For depression, nine studies [22,26,27,29,45,47,48,50,51], comprising 12 independent samples (Table 3), contributed to the meta-analysis (total *n* = 2177). As expected, there was evidence of between-study heterogeneity (Q = 59.43, *p* < .0001). The random effects analysis (GG vs. GA+AA) indicated no evidence of association (Cohen’s *d* = 0.12, 95% CI = [-0.12, 0.36], *Z* = 0.99, *p* = .32) (Fig 4A). Begg’s test (Kendall's tau = 0.09, *p* = 0.74) indicated no evidence of publication bias (Fig 4B), and meta-regression analyses indicated that sex (k = 12, *p* = .70), age (k = 11, *p* = .85), and ethnicity (k = 12, *p* = .86) did not significantly influence the association. In summary, there was no evidence of an association between the polymorphism and depression. Therefore, the genetic association with general sociality was unlikely to be mediated by depression.

**Table 3:** Characteristics of studies on the association between rs53576 polymorphism and depression.

Authors (Year)	Country	Ethnicity	% of Female	Diagnosis	Age (Mean/SD)	Phenotypes	Measurement tools
Bakermans-Kranenburg et al., 2008 [45]	Netherlands	95% Caucasian	100%	Healthy	33 (4.1)	Depression	Short Form of Young Adult Self-Report
Costa et al., 2009 [48]	Italy	100% Caucasian	73%	Bipolar disorder	40.9 (11.7)	Depression	Hamilton Depression Rating Scale
Costa et al., 2009 [48]	Italy	100% Caucasian	68%	Unipolar depression	44.4 (12.5)	Depression	Hamilton Depression Rating Scale
Kawamura et al., 2010 [50]	Japan	100% Asian	38%	Healthy	40.9 (9.7)	Depressive	Temperament Evaluation of Memphis, Pisa, Paris and San Diego
Riem et al., 2011 (sample1) [51]	Netherlands	87.5% Caucasian	100%	Healthy	27.5 (7.6)	Depression	Center for Epidemiological Studies Depression Scale
Riem et al., 2011 (sample2) [51]	Netherlands	87.5% Caucasian	100%	Healthy	27.5 (7.6)	Depression	Center for Epidemiological Studies Depression Scale
Saphire-Bernstein et al., 2011 [22]	U.S.A.	27% Caucasian	61%	Healthy	21.3 (Range:18–36)	Depression	Beck Depression Inventory
Sturge-Appel et al., 2012 [47]	U.S.A.	100% Caucasian	100%	Healthy	28.5 (6.0)	Depression	Computerized Diagnostic Interview Schedule IV
Sturge-Appel et al., 2012 [47]	U.S.A.	100% non-Caucasian	100%	Healthy	25.5 (5.8)	Depression	Computerized Diagnostic Interview Schedule IV
McQuaid et al., 2013 [27]	Canada	58% Caucasian	74%	Healthy	20.0 (3.2)	Depression	Beck Depression Inventory
Wang et al., 2014 [29]	China	100% Asian	53%	Healthy	23.7 (2.5)	Depression	Beck Depression Inventory-II
Bryant et al., 2013 [26]	Australia	100% Caucasian	N.A.	Healthy	N.A.	Depression	Beck Depression Inventory

## Discussion

In this study, we examined the relationship between the polymorphism of a widely-investigated common variant (rs53576) in OXTR and sociality. The meta-analysis on the overall samples revealed that G allele homozygotes were generally more sociable than A allele carriers (Cohen’s *d* = 0.11). In addition, we did not find an association between rs53576 polymorphism and depression, suggesting that its genetic association with general sociality was unlikely to be influenced by depression. On the other hand, somewhat surprisingly, we did not find significant difference in measures of close relationship between G allele homozygotes and A allele carriers. In summary, our study suggested that the rs53576 polymorphism in the OXTR predicts how an individual generally responds to other people, but the polymorphism might be unrelated to individual differences in close relationships (i.e., parent-child or romantic/marital).

Because general sociality behaviors and behaviors in close relationship are functionally distinct constructs, it is necessary to perform separate meta-analyses on these two constructs. Indeed, when these constructs were combined into a single construct, the genetic association between rs53576 polymorphism and this monolith construct was not detected. This result was consistent with that of Bakermans-Kranenburg and van IJzendoorn [16], who performed a similar meta-analysis on the relationship between rs53576 polymorphism and socially related personality traits and behaviors, but did not distinguish general sociality from sociality in the context of close relationships in their analysis.

At first glance, the lack of association between the rs53576 polymorphism and sociality in close relationships appears inconsistent with the popular notion that oxytocin is critical to love [52]. For instance, the mortality rate of offspring increased in oxytocin receptor knockout female mice [53]. Similarly, intranasal oxytocin facilitates recognition of positive sex and relationship words [54]. One possibility is that some OXTR SNP other than rs53576 modulates individual differences in close relationship sociality. For instance, a study has found that individual differences in attachment are associated with the rs2254298 genetic polymorphism, another SNP in OXTR [37].

Our finding of the association between rs53576 polymorphism and general sociality converges with other lines of evidence from the oxytocin literature [3,4,55]. For instance, OXTR knockout mice display deficits in social memory [56] and exhibit autistic-like symptoms [57]. G allele homozygotes (rs53576) have shown greater amygdala activation than A allele carriers when processing socially relevant information [11]. Based on the current literature, future animal and human neuroimaging studies are needed to further explore the exact biological mechanisms that may account for this relationship.

Our study raises a broad question that whether general sociality and close relationships are interrelated. One possibility is that general sociality serves as a basis for other types of sociality, such as close relationships. Another possibility is that these two constructs are completely independent. To solve this question, one might rigorously evaluate the correlations of individual differences in these constructs. The results may be broadly relevant to the search for genetic associations of sociality phenotypes. For example, if there are shared variances between these constructs, it will be more efficient to measure them together when searching for genetic associations. Otherwise, conducting separate genetic association studies on each of them would be sufficient.

Furthermore, several studies have examined the genetic association between the rs53576 polymorphism and depression, and both positive [22] and null results [26, 27, 29, 45, 48, 51] have been reported. By synthesizing these results, the current meta-analysis revealed a lack of association. As we noted previously, depression is often associated with withdrawal from social activities [21]. However, depression also is a state of low mood. Consequently, individual differences in sociality and depression may only share a small portion of variance, and such overlapping may not be affected by the rs53576 polymorphism. Therefore, it is not surprising that the rs53576 polymorphism is only selectively associated with general sociality but not depression.

In addition, several other limitations of the current study deserve consideration. First, the current study is based mainly on results from published studies. Future analyses may include data from more unpublished datasets, such as large-sample databases from genome-wide association studies (GWAS) [58]. Second, compared with many large-sample genetic association studies, the sample size of included studies in our meta-analysis is relatively small which may limit the statistical power of our meta-analysis. Third, interactions between the rs53576 polymorphism and culture (e.g., American vs. East Asian culture) have been suggested to impact sociality [25]. However, we were unable to evaluate this potential interaction in the present study due to the small number of available samples. Fourth, because of a lack of sufficient studies for any single measure of sociality, various measures of general sociality (e.g., extraversion, empathy, and social support seeking) were categorized into a single phenotype. Therefore, we were unable to evaluate possible genetic associations for a specific measure (e.g., empathy). Future studies are needed to test such possible associations in details.

In conclusion, our study provides clear evidence of a relationship between the rs53576 polymorphism and general sociality. In the recent decade, genetic investigation of endophenotypes offers great opportunity as an alternative to investigations of categorical disease phenotypes [59]. It is possible that the general sociality serve as an endophenotype of socially related psychiatric disorders, such as autism and social anxiety disorder. For instance, a recent meta-analysis have revealed that several OXTR SNPs are associated with autism spectrum disorder (ASD) [60], although such an association was not found on rs53576. Potential reasons for the lack of association between rs53576 polymorphism and ASD in that meta-analysis are manifold and include i) a legitimate lack of genetic association, and ii) lack of statistical power (N = 2800, 5 independent samples). Additional studies with adequate methodology are needed to explore the relation between OXTR polymorphism and these socially related psychiatric disorders.

## Supporting Information

S1 File List of excluded articles. (DOCX)

S1 Table PRISMA 2009 Checklist. (DOC)

S2 Table Meta-analysis on Genetic Association Studies Checklist. (DOCX)
