# Supplementary material for: Association of Oxytocin Receptor Gene (OXTR) rs53576 Polymorphism with Sociality: A Meta-Analysis
Source: PLoS One. 2015 Jun 29;10(6):e0131820. doi: 10.1371/journal.pone.0131820 (PMC4488068; doi:10.1371/journal.pone.0131820)
Supplement: S1 File — (DOCX) [file pone.0131820.s001.docx]

List of Excluded Articles

**Duplicate sample**

Kim HS, Sherman DK, Mojaverian T, Sasaki JY, Park J, Suh EM, et al. Gene-Culture Interaction: Oxytocin Receptor Polymorphism (OXTR) and Emotion Regulation. Soc Psychol Personal Sci. 2011; 2: 665–672.

Sasaki JY, Kim HS, Xu J. Religion and Well-being. J Cross-Cult Psychology. 2011; 42(8): 1394-1405.

(included article: Kim HS, Sherman DK, Sasaki JY, Xu J, Chu TQ, Ryu C, et al. Culture, distress, and oxytocin receptor polymorphism (OXTR) interact to influence emotional support seeking. Proc Natl Acad Sci USA. 2010; 107: 15717–15721.)

Lucht MJ, Barnow S, Sonnenfeld C, Ulrich I, Grabe HJ, Schroder W, et al. Associations between the oxytocin receptor gene (OXTR) and "mind-reading" in humans-An exploratory study. Nordic Journal of Psychiatry, 2013; 67(1): 15-21

(included article: Lucht MJ, Barnow S, Sonnenfeld C, Rosenberger A, Grabe HJ, Schroeder W, et al. Associations between the oxytocin receptor gene (OXTR) and affect, loneliness and intelligence in normal subjects. Prog Neuropsychopharmacol Biol Psychiatry. 2009; 33: 860–866)

Kawamura, unpublished data

(included article: Kawamura Y, Liu X, Shimada T, Otowa T, Kakiuchi C, Akiyama T, et al. Association between oxytocin receptor gene polymorphisms and autistic traits as measured by the Autism-Spectrum Quotient in a non-clinical Japanese population. Asia-Pacific Psychiatry. 2011; 3: 128–136)

**Lack of data**

Apicella CL, Cesarini D, Johannesson M, Dawes CT, Lichtenstein P, Wallace B, et al. No Association between Oxytocin Receptor (OXTR) Gene Polymorphisms and Experimentally Elicited Social Preferences. PLoS ONE. 2010; 5(6): e11153.

Bradley B, Westen D, Mercer KB, Binder EB, Jovanovic T, Crain D, et al. Association between childhood maltreatment and adult emotional dysregulation in a low-income, urban, African American sample: Moderation by oxytocin receptor gene. Dev Psychopathol. 2011; 23(02): 439-452.

Montag C, Brockmann EM, Lehmann A, Muller DJ, Rujescu D, & Gallinat J. Association between Oxytocin Receptor Gene Polymorphisms and Self-Rated 'Empathic Concern' in Schizophrenia. PLoS ONE. 2012; 7(12): e51882.

Montag C, Sauer C, Reuter M, & Kirsch, P. An interaction between oxytocin and a genetic variation of the oxytocin receptor modulates amygdala activity toward direct gaze: evidence from a pharmacological imaging genetics study. Eur Arch Psychiatry Clin Neurosci. 2013; 263(2): 169-175.

Poulin MJ & Holman EA. Helping hands, healthy body? Oxytocin receptor gene and prosocial behavior interact to buffer the association between stress and physical health. Horm Behav. 2013; 63(3): 510-517.

Bradley B, Davis TA, Wingo AP, Mercer KB, & Ressler KJ. Family environment and adult resilience: contributions of positive parenting and the oxytocin receptor gene. Eur J Psychotraumatol. 2013; 4: 21659.

Mendlewicz J, Crisafulli C, Calati R, Kocabas NA, Massat I, Linotte S, et al.. Influence of COX-2 and OXTR polymorphisms on treatment outcome in treatment resistant depression. Neurosci Lett. 2012; 516(1): 85-88.

Tabak BA, McCullough ME, Carver CS, Pedersen EJ, & Cuccaro ML. Variation in Oxytocin Receptor Gene (OXTR) Polymorphisms is Associated with Emotional and Behavioral Reactions to Betrayal. Soc Cogn Affect Neurosci. 2014; 9(6):810-816.

Lucas-Thompson RG & Holman EA. Environmental stress, oxytocin receptor gene (OXTR) polymorphism, and mental health following collective stress. Horm Behav. 2013; 63(4): 615-624.
